# Supplementary material for: Physical Activity, Sport and Physical Education in Northern Ireland School Children: A Cross-Sectional Study
Source: Int J Environ Res Public Health. 2020 Sep 19;17(18):6849. doi: 10.3390/ijerph17186849 (PMC7559058; doi:10.3390/ijerph17186849)
Supplement: Supplementary file 1 [file ijerph-17-06849-s001.pdf]

Supplementary material

Table S1. Outcome Measures.

Measure	Description	Source	Validity
**Child Functioning Module**	24 item questionnaire which assesses functional difficulties across domains including hearing, vision, communication, comprehension, learning, mobility and emotions on a rating scale (no difficulty, some difficulty, a lot of difficulty or cannot do at all)	Cappa et al. (2018) [33] Loeb et al. (2017) [34]	Validity assessed by Sprunt et al. (2019) for use by proxy by parents and teachers. Inter-rater reliability was 0.68 (95% CI 0.60–0.73).
**Family Affluence Scale**	4 item measure of parental socio-economic status for adolescents	WHO Health Behaviour in School-aged Children Study	For the composite FAS II index, there was a strength in rank order correlation (0.87) with Gross Domestic Product (GDP) and a Kappa agreement coefficient of 0.57, indicating good criterion validity of FAS II (Boyce et al., 2006)
**Physical Activity Enjoyment Scale (PACES)**	16-item questionnaire that asks respondents to complete statements pertaining to why they may enjoy PA (e.g. "I get something out of it"), using a 5-point Likert scale ranging from 1 (Disagree a lot) to 5 (Agree a lot).	Motl et al. (2001) [39]	Validated to measure physical activity enjoyment in primary school children (Woods et al., 2012), in adolescent girls (Motl et al., 2001) and in older adolescents (Dunton et al., 2009). Structural equation modeling which consisted of 4 latent variables (enjoyment, factors influencing enjoyment of PE, PA and sport involvement) was tested using confirmatory factor analysis. CFA indicated that the measurement model represented an acceptable fit (X^2 = 1769.57, df = 451, RMSEA = 0.040 [90% CI = 0.038–0.042], RNI = 0.93, NNFI = 0.92). Interfactor correlations were significant and ranged between 0.19 and 0.45 (M = 0.30, Md = 0.28) (Motl et al., 2001). Internal consistency was α = 0.87 when validated on primary school children (Woods et al., 2012).
**PACE+**	2 item screening tool—1) number of days active for at least 60 mins in the past 7 days and 2) number of days active for at least 60 mins in a typical week	Hardie-Murphy et al. (2015) [37]	Shown to have acceptable validity and reliability for assessing adherence to PA guidelines (Hardie-Murphy et al., 2015; Ridgers et al., 2012; [38] Vuori et al. (2005); Prochaska et al., 2001). Spearman correlations between self-reported physical activity levels and accelerometry derived minutes of moderate-to-vigorous physical activity per day were small (r = 0.27; seven valid days) to moderate (r = 0.34; ≥5 valid days). The accuracy of classifying those not meeting the guidelines (specificity) was moderate to high (59–100%). Intraclass correlation coefficients (ICC) ranging from 0.6 to 0.8
**Economic and Social Research Institute (ESRI) survey**	Participants reported the frequency and duration of PE classes in curriculum time and frequency of participation in school sport (school-based sport outside of curriculum time) and community sport (in non-school clubs) from the following options "4 or more days a week", "2–3 days a week", "once a week", "2–3 days a month", "one day a month", "less often" and "never". Participants were categorised as a 'current participant' in school sport or community sport if they participated in either domain at least once per week.	Fahey et al. (2005) [40]	Unvalidated
**Social Support**	The 5 item family social support scale requires the participant to indicate how often a member of their family encouraged them to do PA, did PA with them, provided transportation to a place to do PA, watched them do PA or told them that that were doing well in PA or sport on a 6 point likert scale ranging from never (0) to everyday (5). Scores on each item are then added for a minimum score of 0 and a maximum score of 25. The peer measure asked the respondent to indicate frequency of encouraging friends to do PA, encouragement from friends to do PA, doing PA with friends, being teased by friends for not being good at PA (reversed scoring) and being told that you are doing well in PA or sport. The same likert scale and scoring system was used.	Amherst Health and Activity Study [41]	Psychometric properties of social support scales show strong internal consistencies for family (α = 0.78, ICC 0.81) and peers (α = 0.74, ICC 0.70) Sallis et al. (2002)
